# A parametric neutron Bragg edge imaging study of additively manufactured samples treated by laser shock peening

**DOI:** 10.1038/s41598-021-94455-3

**Published:** 2021-07-21

**Authors:** Matteo Busi, Nikola Kalentics, Manuel Morgano, Seth Griffiths, Anton S. Tremsin, Takenao Shinohara, Roland Logé, Christian Leinenbach, Markus Strobl

**Affiliations:** 1 Paul Scherrer Institute, Laboratory for Neutron Scattering and Imaging, Forschungsstrasse 111, 5232 Villigen, Switzerland; 2 Thermomechanical Metallurgy Laboratory—PX Group Chair, Ecole Polytechnique Fédérale de Lausanne (EPFL), 2002 Neuchâtel, Switzerland; 3 Empa-Swiss Federal Laboratories for Materials Science and Technology, 8600 Dübendorf, Switzerland; 4 University of California, Berkeley, CA 94720 USA; 5 J-PARC Center, Japan Atomic Energy Agency, Tokai, Ibaraki 319-1195 Japan

**Keywords:** Mechanical properties, Characterization and analytical techniques, Imaging techniques

## Abstract

Laser powder bed fusion is an additive manufacturing technique extensively used for the production of metallic components. Despite this process has reached a status at which parts are produced with mechanical properties comparable to those from conventional production, it is still prone to introduce detrimental tensile residual stresses towards the surfaces along the building direction, implying negative consequences on fatigue life and resistance to crack formations. Laser shock peening (LSP) is a promising method adopted to compensate tensile residual stresses and to introduce beneficial compressive residual stress on the treated surfaces. Using neutron Bragg edge imaging, we perform a parametric study of LSP applied to 316L steel samples produced by laser powder bed fusion additive manufacturing. We include in the study the novel 3D-LSP technique, where samples are LSP treated also during the building process, at intermediate build layers. The LSP energy and spot overlap were set to either 1.0 or 1.5 J and 40$\%$ or 80$\%$ respectively. The results support the use of 3D-LSP treatment with the higher LSP laser energy and overlap applied, which showed a relative increase of surface compressive residual stress (CRS) and CRS depth by 54$\%$ and 104$\%$ respectively, compared to the conventional LSP treatment.

## Introduction

Laser powder bed fusion (LPBF), also known as selective laser melting (SLM), is a widely used technique for additive manufacturing (AM). Using a high-intensity laser, LPBF produces metallic parts by melting consequent layers of metallic powders following predefined models. Its applications can be found in many fields, including medical^1–3^, aerospace^4^, turbines^5^, robotics^6^ and in particular for the production of unique and highly complex components^7^. Being the overarching goal, the optimization and enhancement of this technique have seen a rapid growth and have been supported by intense research for years. While this technique has already reached a status in which the mechanical properties of materials produced are comparable to those from conventional production processes, LPBF still has several limitations. Among those, especially the accumulation of detrimental tensile residual stress (TRS) is critical in the proximity of the final layer built, due to shrinkage processes occurring during the solidification of the molten powder. The presence of TRS in exposed parts of the materials can result in delamination, distortions, reduced fatigue life or even cracking that can occur during the building phase^8^.

While several techniques, such as in-situ heating or annealing, have been used and proposed to limit or reduce the formation of TRS at the sample surface, these are not able to induce beneficial residual stresses, i.e. compressive residual stresses (CRS), which improve dramatically fatigue life and the material’s resilience to external forces^9^. Laser shock peening (LSP) is a surface treatment method that has been developed to counteract the presence of TRS. It has been shown that LSP has the ability to push surface TRS deeper into the sample and introduce in turn CRS in the surface region^10^. Recently, a new technique called 3D laser shock peening (3D-LSP) was developed, which integrates the LSP treatment in the LPBF process at multiple selected layers, which typically have a thickness of 30 to 70 micrometers. Kalentics et al.^11^ have shown that with 3D-LSP, the heat induced by the LPBF layer building is not generally enough to cause the relaxation of the CRS introduced by the LSP treatment in the previous layer. Hence, the values of surface CRS and respective depth can be dramatically improved, compared to conventional surface LSP. Furthermore, it is possible to control the depth of the CRS introduced by adjusting the laser energy and spot overlap, i.e. number of shots per surface area. The effect of LSP could initially be characterized locally by the incremental hole-drilling method^12,13^, however, this method is highly local and destructive and is only accurate in the investigation of stresses up to about a millimeter depth into the sample. Among the nondestructive methods, neutron diffraction and the X-ray diffraction (XRD) are widely used however, XRD is in general limited to surface analyses due to the limited penetration depth in many metals and neutron diffraction techniques are typically limited in spatial resolution.

To overcome these limitations, neutron imaging methods based on diffraction contrast^14^ have been introduced. Thanks to the nature of neutron-matter interactions, such as the high penetration depths of neutrons in many relevant elements and compounds, these methods can be efficiently used to investigate the bulk of metallic components. Large volumes can thus be assessed with spatially resolved single exposure measurements providing information of local density, strains^14–16^, phase composition^14,17^ and texture variations^18,19^. With the advent of novel pixelated time-of-flight (TOF) detectors, which enable wavelength resolved imaging measurements, neutron Bragg imaging was demonstrated both in 2D, in the form of projection imaging^14,16,17^, and 3D in the form of tomography^14,20–22^ and in time resolved in-situ studies^23^. In a recent study, we have applied Bragg edge imaging to assess the residual stress introduced in additively manufactured steel samples by post processing treatments such as laser shock peening (LSP)^24^. Therein, we demonstrated the efficiency of the method by comparisons with the conventional hole-drilling method, in particular in the surface regions and with spatial resolutions down to about 55 $\upmu$m pixel size. Bragg edge imaging was also used to efficiently study the influence of multiple LPBF processing parameters on the resulting TRS at the sample surfaces^25^.

In this work, we present a study of the induced residual stress fields in stainless steel 316L samples depending on the processing parameters adopted in the LPBF process and in particular the novel 3D-LSP treatment, as well as a comparison with conventional post process LSP treatment. Utilizing neutron Bragg edge imaging, we characterized a relatively large series of samples through efficient full field single shot measurements. The influence of the printing and 3D-LSP treatment conditions on the residual stress in the samples was analyzed with a specific focus on the quantification of the magnitude and depth of the CRS introduced by the LSP treatments.

## Methods

Neutron Bragg edge imaging, differently from conventional attenuation-based neutron imaging methods, in which the measured signal is the integral of a polychromatic beam, enables access to the wavelength dependence of the attenuation coefficient with spatial resolution. Depending on the material and the wavelength bandwidth of the source spectrum, additional information on the crystalline properties of the samples can be extracted. Wavelengths for which the elastic coherent scattering dominates the total cross sections provide information on crystallographic properties such as strain (hence, residual stress), texture and crystalline phase composition. Additionally, longer wavelengths, where the dominant interaction is the neutron absorption, provide a more accurate representation of the bulk density distribution, which is not contaminated by texture variations within the samples.

**Figure 1:**
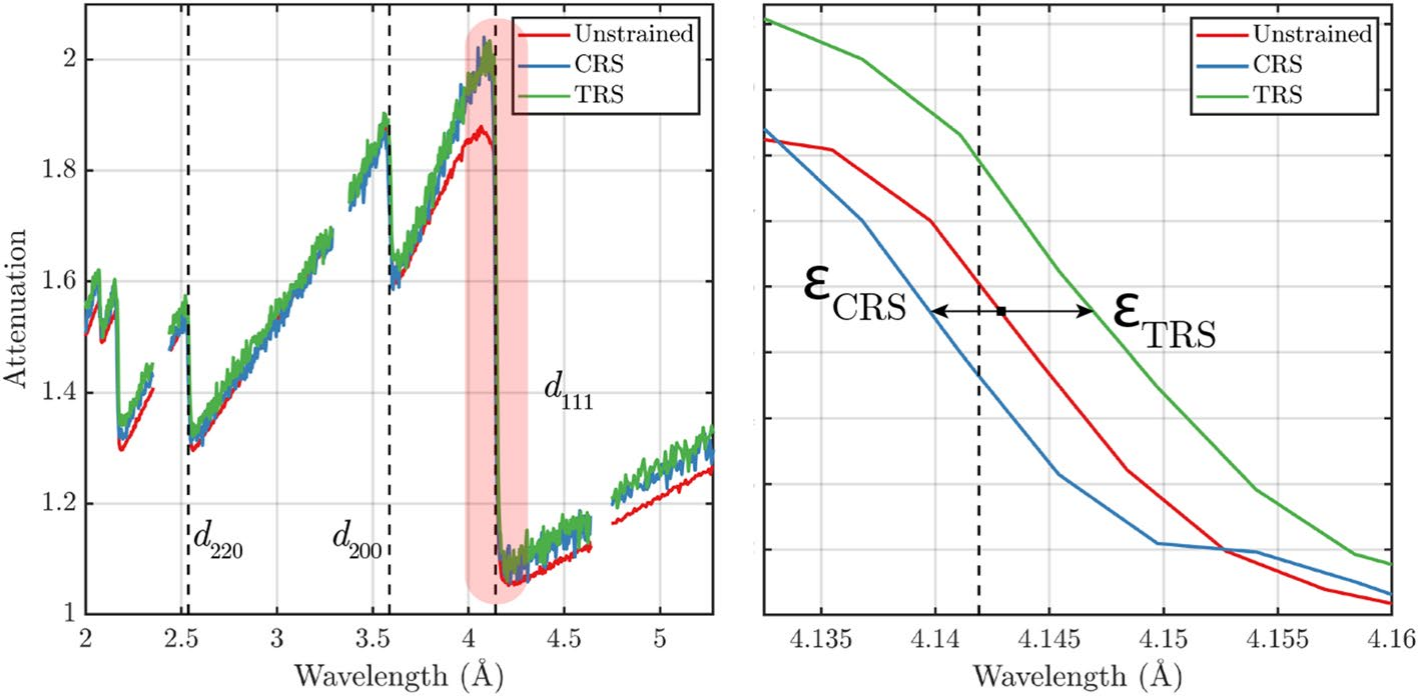
Spectra of the wavelength dependent linear attenuation coefficient for the unstrained annealed sample (red) and two sample zones with CRS (blue) and TRS (green), showing the Bragg edges corresponding to the lattice planes (220), (200) and (111). The right frame magnifies the wavelength region corresponding to the (111) lattice planes. The relative deviation of each of the Bragg edge wavelength positions is converted to elastic strain $\epsilon _{\mathrm {hkl}}$.

Figure 1 shows the attenuation coefficient spectra of the unstrained stainless steel, a sample that has been annealed, and two selected regions of another sample displaying CRS and TRS, respectively. In the figure, discontinuities in the attenuation spectra are visible at approximately 2.6 $\r{A}$, 3.6 $\r{A}$ and 4.1 $\r{A}$. These are the so called Bragg edges and the localization of their respective wavelength $\lambda _{\mathrm {hkl}}$ can be used to calculate the corresponding lattice spacing as $d_{\mathrm {hkl}} = \lambda _{\mathrm {hkl}}/2$ from the Bragg’s condition for backscattering $\lambda = 2 d_{\mathrm {hkl}}\sin {\theta }$, where $d_{\mathrm {hkl}}$ is the lattice planes spacing of a specific family of lattice planes (${\mathrm {hkl}}$) and the scattering angle $\theta$ is set to $\theta = \pi /2$. The deviations of the measured lattice spacing from a reference unstrained lattice spacing $d^0_{\mathrm {hkl}}$ can thus, be used to calculate the elastic strain (see Fig. 1), $\epsilon _{\mathrm {hkl}}$, according to the equation:

1 $$\begin{aligned} \epsilon _{\mathrm {hkl}} = \frac{d_{\mathrm {hkl}}-d^0_{\mathrm {hkl}}}{d^0_{\mathrm {hkl}}}, \end{aligned}$$

In the case of neutron Bragg edge imaging, for thick samples, the measured strains correspond to the average strain over the sample thickness in the beam’s direction. The residual stress, $\sigma _{\mathrm {hkl}}$ (MPa), can then be calculated when assuming linear elasticity, and a specific Young’s modulus, *E*, using the equation:

2 $$\begin{aligned} E = \sigma _{\mathrm {hkl}}/\epsilon _{\mathrm {hkl}}. \end{aligned}$$

The Young’s modulus for stainless steel 316L and the considered lattice plane (111), was assumed to be 261 GPa. This value was calculated from strain-stress scatter plots for similar samples of stainless steel 316L^26^.

In this work, the evaluation of the lattice spacing used to calculate the elastic strain and stress according to Eqs. (1) and (2), is performed by a first order Gaussian fit of the derivative of the attenuation coefficient. The centroid of the Gaussian curve is used to determine the wavelength of the Bragg edge, from which the lattice spacing is obtained. In this work, this routine was applied for each pixel of the 2D radiographs of each sample and for a wavelength bandwidth containing the FCC (111) Bragg edge at approximately 4.1 $\r{A}$, as it represents the strongest variations in the attenuation coefficient. All the radiographs have been convoluted with a one-dimensional moving average kernel, with windowing size 5 and in the direction orthogonal to the build direction, to increase the neutron statistics prior to the Beer-Lambert’s normalization of the signal into attenuation coefficient. The reference lattice spacing $d^0_{\mathrm {hkl}}$ was obtained from a sample that was annealed after the AM build process.

Morgano et al.^24^ validated this method in a previous work with the hole drilling method (HDM) by comparing measures strains in the surface region of identical samples from both Bragg edge imaging and HDM. The results have shown good agreement, but also underlined the higher resolution of HDM at the surface and better accuracy of Bragg edge imaging beyond about 1 mm depth under the surface, where HDM becomes unreliable^27^. In the present work, we further convert the strain to residual stress using Eq. (2), and our results reported in the next sections are in agreement with the literature range of values for additively manufacturing stainless steels^11,28,29^.

## Experimental setup

### Instrumentation

The measurements were carried out at the neutron beam line RADEN at the J-PARC pulsed neutron spallation source in Japan. Shinohara et al. presented latest development and instrumentation details of the beam line^30^. RADEN has instrumentation that allows for time-of-flight neutron imaging experiments, with neutron wavelengths that reach up to 8.8 $\r{A}$ with a resolution of approximately $\Delta \lambda /\lambda = 0.2\%$ above 3 $\r{A}$. The detector used was a pixelated micro-channel plate (MCP)/Timepix detector and its latest developments are reported Tremsin et al.^31^. This detector has a pixel size of 55 $\upmu {\mathrm {m}}$, and $512\times 512$ pixels. The corresponding field of view is $28.16\times 28.16$ ${\mathrm {mm}}^2$, allowing for the simultaneous imaging of multiple samples. Dead-time losses were corrected using a standard correction algorithm, which was presented by Tremsin et al.^32^. For our measurements, we selected a wavelength bandwidth between 1.5 $\r{A}$ to 5.3 $\r{A}$, with TOF binning width of 0.004 $\r{A}$ so that the instrumental resolution of 0.2$\%$ was sampled with two points. This was chosen to suit the analysis of the most pronounced Bragg edges for the lattice planes (220), (200) and (111), visible in Fig. 1. The exposure time for each set of samples was of 4 hours.

**Figure 2:**
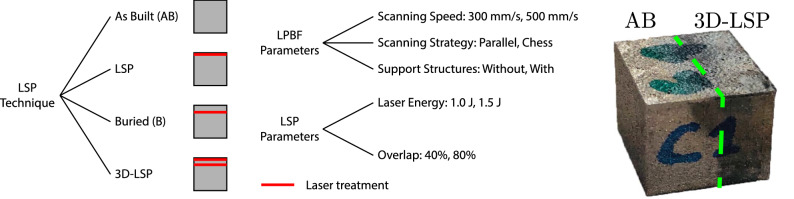
Diagram sketch of different production parameters used to build the samples and a picture of one specimen, where the chess LPBF scanning pattern is visible and the dark area corresponds to the part of the sample that has been treated with LSP.

### Materials

The samples processed in this work are processed from the same batch of samples from our previous publication^25^, as some of the LPBF parameters are identical. These are rectangular cuboids with dimensions of approximately $12\times 14$ $\hbox {mm}^2$ cross section and a building height of approximately 10 mm. The samples were manufactured using MetcoAdd spherical powder (Oerlikon Metco, Switzerland) of 316L austenitic stainless steel (i.e. Fe-17Cr-12Ni) with mean diameter 31.86 $\upmu {\mathrm {m}}$ and a Concept M2 machine (Concept Laser GmbH, Germany). The machine is equipped with a fiber laser operating in continuous mode with a Gaussian intensity distribution and a wavelength of 1070 nm and a spot size (1/$\hbox {e}^2$) of 90 $\upmu {\mathrm {m}}$. The laser energy, hatch distance and powder layer thickness were kept fixed to 125 W, 105 $\upmu {\mathrm {m}}$ and 30 $\upmu {\mathrm {m}}$ respectively. The samples were produced under ${\hbox {N}_2}$ atmosphere and the ${\hbox {O}_2}$ content was kept below 1$\%$ during the process.

**Table 1:** Sample labels corresponding to the different LPBF parameter combinations, and the listing of the sample measured for each LSP technique.

LPBF Parameters								
Sample series:	A	B	C	D	E	F	G	H
Laser speed:	High	High	High	High	Low	Low	Low	Low
Strategy:	Parallel	Parallel	Chess	Chess	Parallel	Parallel	Chess	Chess
Support structure:	✗	✓	✗	✓	✗	✓	✗	✓

Buried								
Overlap	Energy							
	1.0 J	1.5 J						
40$\%$	B, E	A, $\hbox {C}^*$						
80$\%$	$\hbox {G}^*$, D	F, $\hbox {H}^*$						

LSP								
Overlap	Energy							
	1.0 J	1.5 J						
40$\%$	$\hbox {A}^*$, B, C	E, F, G, H						
80$\%$	/	$\hbox {A}^*$, B, C, D, E, F, G, H						

3D-LSP								
Overlap	Energy							
	1.0 J	1.5 J						
40$\%$	/	D, E, F, G, H						
80$\%$	$\hbox {A}^*$, B, C, $\hbox {D}^*$	A, $\hbox {B}^*$, C, D, E, F, G, H						

Figure 2 details different processing parameters adopted to produce the samples measured in this study. The build procedures differ in the modalities in which the LSP treatment was applied. The samples were either left in the as built condition (AB) or were treated with different LSP strategies. In conventional LSP, only the topmost layer of the sample was treated (LSP). In the buried (B) and 3D-LSP case, the LPBF process was interrupted 20 layers before the end. Then, the LSP treatment was applied, and then the LPBF layers build was continued. For the 3D-LSP case, additionally, the final layer was treated with LSP. For each of the LSP treatment strategies listed, the LPBF and LSP processing parameters were varied resulting in a multiplicity of parameter combinations. At the actual state of the technique, an integrated machine that combines the two processes was not available and the samples were moved from the LPBF machine to the LSP treatment and back by interrupting the printing process at specific layers.

The LPBF processing parameters were the laser scanning speed, which was set to either *low*- or *high*-speed corresponding to 300 and 500 mm/s respectively, and were built *with* or *without* 3 mm thick support structures. In all cases, the samples were cut via electro-discharge machining in order to have the same sample height, where in the first case the support structure was cut and in the second case additionally printed bottom layers of the same material exceeding the target sample height were cut. Finally, the laser scanning strategy was modified. In the *parallel* mode, the powder layers are melted without a change in the scanning orientation within the same layer, but alternating laser directions rotated by 90 degrees at each layer. In the *chess* method, two orthogonal scanning orientations are alternated in a chessboard pattern within the same layer. The laser wavelength was 1064 nm, with a laser spot size of 1 mm and pulse duration of 6.3 ns. Based on results obtained in previous studies^10^, the laser energy used for the LSP treatment was set either to 1.0 J or to 1.5 J, corresponding to a laser density of 20 GW/$\hbox {cm}^2$ and 30 GW/$\hbox {cm}^2$ respectively. Finally, the laser shots overlap, i.e. the amount of overlap between two successive laser shock pulses applied to the surface, was either 40$\%$ or 80$\%$. Figure 2 shows a picture of a specimen built with chess laser scanning strategy, where half of the sample was treated with 3D-LSP and the other half left AB.

Table 1 represents the sample labels according to each LPBF parameter combinations. For each of these 8 labels, a selection series of 6 samples, in as built conditions and with different LSP technique and parameters were placed in the neutron beam with an exposure time of 4 hours. The total number of samples measured was 48 however, some of the corresponding datasets (denoted by the asterisk) had to be discarded due to finding a failure of the LSP treatment determined *a posteriori*, by the analysis of the residual stress curves and confirmed by visual inspection of the samples. Table 1 also lists, for each of the LSP techniques and process parameters, the LPBF labels respective to the samples measured.

## Results and discussion

**Figure 3:**
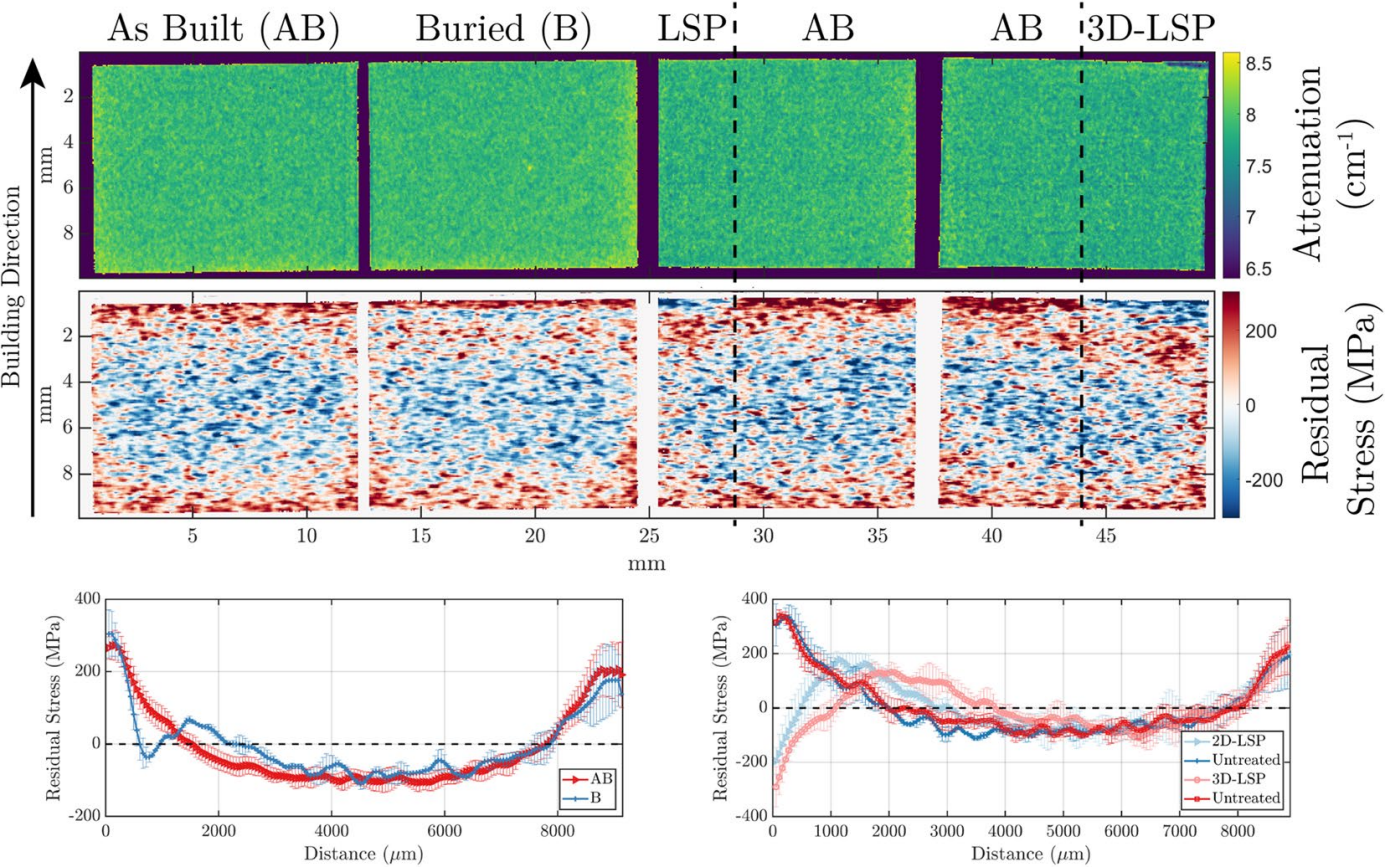
Top: attenuation-based contrast map of four samples exposed to different treatments. Center: the respective residual stress map. Bottom: Residual stress, measured along the build direction, of the four samples, integrated over selected regions. The RS lines corresponding to the AB and B samples were integrated over all the sample area whereas the LSP and 3D-LSP were split in two regions delimited by the black dash-dotted lines, as partial volumes of these samples were left untreated.

Figure 3 shows density maps according to the attenuation for wavelengths above 4.2 $\r{A}$ and stress maps corresponding to the (111) lattice planes for a selection of samples, one for each LSP technique. The AB and B samples were built with parallel laser scanning strategy (series A) whereas the LSP and 3D-LSP specimens were built with chess strategy (series C), and they were all built with high speed and no support structures.

Note that sample surfaces were treated with LSP only partially, also in case of the buried “surfaces” of buried LSP and 3D-LSP. The treated regions can easily be identified in the residual stress maps by the presence of CRS near the top surface, which allows direct comparisons between a treated and untreated part of the same sample. In addition, the figure presents line profiles of the residual stress along the build direction. Line profiles are averaged for corresponding regions perpendicular to the build direction for each sample. These profiles illustrate the different impact of the LSP treatment techniques with respect to induced CRS in the samples. The sample in AB conditions displays the typical zones of high TRS in the proximity of the surfaces in particular concerning the build direction. The conventional LSP treatment introduces CRS at the (top) surface of the sample. With increasing depth, the induced CRS decreases and eventually turns to TRS, exceeding the initial one at this depth, until the stress follows the same trend as in the AB sample deeper in the bulk. The sample treated with buried LSP shows a similar behavior that differs from the as built mainly in a confined zone of CRS between approximately 500 and 900 $\upmu$m depth under the surface, which is introduced by the LSP treatment at the corresponding depth under the final surface. Increased TRS is found in the adjacent layers. These results indicate and confirm that the heat induced by the LPBF processing of the successive layers, is insufficient to fully relax the CRS introduced by the LSP treatment in the “buried layer”. With the 3D-LSP treatment, the CRS can be extended deeper, along with the TRS zone being pushed deeper into the sample. This further confirms that with the 3D-LSP processing, the CRS introduced by the LSP treatment in a layer can merge with the CRS introduced in the following, LSP treated layer, resulting in an overall higher CRS amplitude and depth. The analysis of the Bragg edge maps, which can be used to detect spatially resolved microstructure and texture variations^14,25^, did not exhibit influence of the LSP on the microstructure, in agreement with previous studies performed with EBSD^10^.

**Figure 4:**
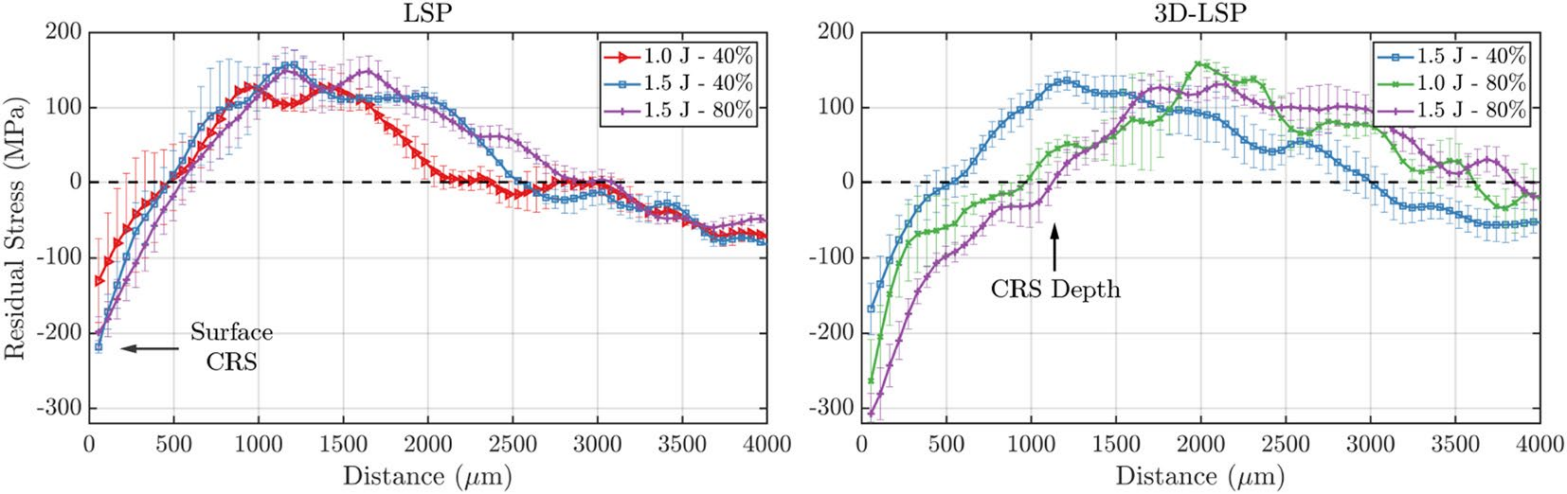
Average residual stress (MPa) as a function of distance from the top sample surface for different LSP process parameters. The plots are grouped depending on whether they are treated by conventional LSP (left) or 3D-LSP (right). The color code and marker style are unique to each specific LSP process parameters.

In the remainder of the section, we report and discuss the results in the form of residual stress line profiles averaged along the build direction as a function of the distance from the top surface. Figure 4 shows such residual stresses along the build direction for multiple combinations of LSP process parameters. These curves were calculated as the average residual stress for all the samples with different LPBF parameters and fixed process parameters (see Table 1). The main characteristics displayed in these maps, that are relevant to the mechanical properties of the material, are (i) the surface CRS, which here is defined as the magnitude of the beneficial compressive residual stresses in the first spatial bin at the surface of the sample (i.e. 55 $\upmu$m), and (ii) the CRS depth, which is the depth where the CRS converts into TRS. While the former quantifies the material resistance to the formation of cracks induced through stresses applied externally, the latter controls the speed of the crack propagation so that deeper CRS result in better fatigue resistance and longer fatigue life. For both LSP and 3D-LSP techniques, the best results are obtained with either 1.5 or 1.0 J laser energy and 80$\%$ spot overlap, while the worst are obtained with 40$\%$ (Fig. 4). This indicates that the density of laser shots is more influential than the laser energy, in terms of resulting CRS. With the 80$\%$ overlap setting, the number of shots per surface area is increased by a factor of 9 compared to the 40$\%$ overlap. Furthermore, it is observed, e.g. in Fig. 4, that with increasing overlap, the TRS is pushed deeper below the surface, and there is, thus, a deeper region of CRS.

**Table 2:** Surface CRS (MPa) measured at 55 $\upmu$m and CRS depth ($\upmu$m) for specific process parameters of the LSP and 3D-LSP treatments.

Surface CRS (MPa)				
Process parameters	1.0 J − 40$\%$	1.5 J − 40$\%$	1.0 J − 80$\%$	1.5 J − 80$\%$
LSP	−130.4	−218.1	/	−198.9
3D-LSP	/	−167.7	−263.3	−306.8

CRS depth ($\upmu$m)				
Process parameters	1.0 J − 40$\%$	1.5 J − 40$\%$	1.0 J − 80$\%$	1.5 J − 80$\%$
LSP	467	467	/	550
3D-LSP	/	550	960	1125

Table 2 details the surface CRS and respective CRS depth for each of the curves corresponding to the LSP process parameters. As expected from previous studies^11^, both increasing the laser energy and the overlap correspond to higher surface CRS and deeper CRS, dominated however, by the larger impact from the overlap. For the samples treated with 1.5 J laser energy and 80$\%$ overlap, the 3D-LSP yields the best results and outperforms the conventional LSP, as the surface CRS and CRS depth have respectively a relative increase of 54$\%$ and 104.5$\%$ compared with conventional LSP. In turn, when the laser energy is kept at 1.5 J but the overlap is decreased to 40$\%$, 3D-LSP still yields deeper CRS (+18$\%$) but potentially lower surface CRS (-23$\%$) compared to the conventional LSP. This is likely to be because for a 40$\%$ overlap, the number of LSP shots per area is insufficient to introduce CRS at the buried layer, which can merge with the CRS from the final surface LSP treatment. The CRS introduced at the buried layer are partially relaxed by the subsequent LPBF rebuilding step, and the benefit of the 3D-LSP compared to conventional LSP in this case would be observable only if the number of rebuilt layers was adequately small such that the CRS from the two LSP processes can merge. Not surprisingly, the sample treated with 1.0 J and 40$\%$ conventional LSP yields the worst values of both surface CRS and CRS depth. As mentioned in the previous section, for some of the samples the LSP treatment process failed and hence, these are missing from the result table (Table 2). However, we observe that for all the samples treated with 80$\%$ overlap, the 3D-LSP clearly outperforms the conventional LSP.

**Figure 5:**
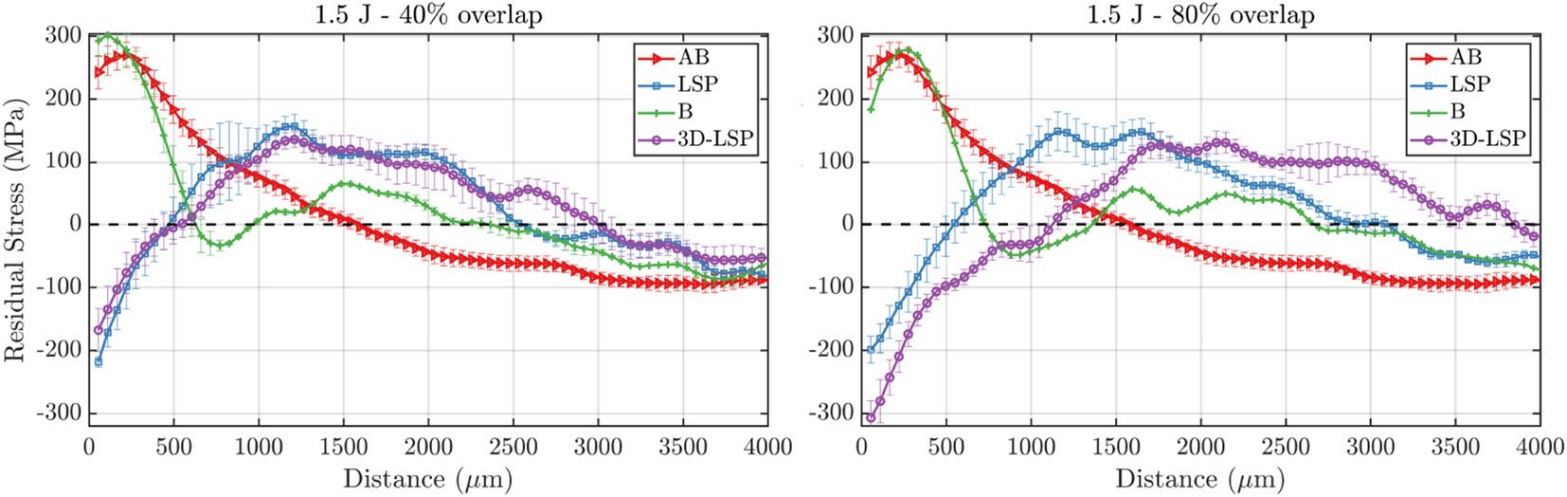
Average residual stress (MPa) as a function of distance from the top sample surface for different LSP techniques. The LSP process parameters were fixed to 1.5 J and 40$\%$ overlap in the left frame and 1.5 J and 80$\%$ overlap in the right frame. The color code and marker style are unique to each specific LSP technique.

Figure 5 illustrates the impact of each LSP approach on the stresses profile, for 1.5 J LSP laser energy and 40$\%$ overlap in the left panel of the figure and 80$\%$ overlap in the right panel. In agreement with the results displayed in Fig. 3, the profiles in both panels confirm the typical trends for the 4 different LSP treatment approaches. For both LSP laser overlap settings the 3D-LSP leads to greater CRS depths compared to the conventional surface LSP, however, the surface CRS is more pronounced only for the 80$\%$ overlap case. The samples built with buried LSP show clearly a zone of CRS confined relatively deep, i.e. a few 100 $\upmu$m up to more than 1 mm, under the surface. Furthermore, it is observed that the depth at which the TRS converts to CRS for buried LSP samples is almost identical to the CRS depth of the respective 3D-LSP treated sample. This indicates that the initial buried treatment was sufficiently effective to compensate the tensile strain effect of the subsequent surface treatment. However, it is further observed that with the LSP settings of 1.5 J and 40$\%$ overlap, for the buried treatment alone, the surface TRS appear slightly higher than for the untreated AB sample (Fig. 5 left), which might be interpreted as a compensation for the buried CRS. This is compatible with the observation that for such LSP settings the results of the 3D-LSP yield worse behavior at the surface in terms of induced CRS than the LSP treatment on the surface alone. However, it has to be noted, that these differences must appear small in particular with regards to the steep strain gradients and the limited spatial resolution. This underlines the importance of the chosen 3D-LSP parameters, and especially the laser overlap and the number of rebuilt layers, for designing a specific stress field and achieving in particular a targeted CRS layer thickness through 3D-LSP.

**Figure 6:**
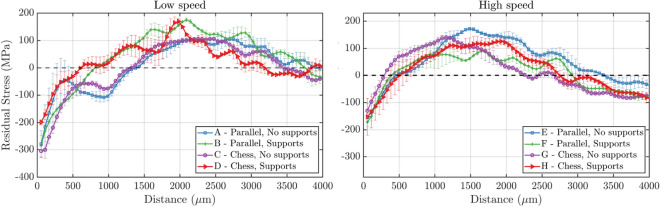
Average residual stress (MPa) as a function of distance from the top sample surface for different LPBF processing parameters. The plots are grouped by LPBF scanning speed set to 300mm/s (left) and 500mm/s (right). For both graphs, the LSP technique was 3D-LSP with 1.5 J laser energy and 80$\%$ spot overlap. The color code and marker style are unique to each specific LPBF processing parameters.

While in the previous comparisons, the samples were chosen with specific LPBF processing parameters to avoid the dependence of the resulting residual stress on these factors, Fig. 6 shows the average residual stress profiles along the build direction for samples built with different LPBF processing parameters. In the figure, the LSP process parameters with which the samples were treated were set to 1.5 J and 80$\%$ overlap and the results are only shown for the 3D-LSP treatments. For both of the two scanning speeds, the LPBF processing parameters yielding the best surface CRS and respective depth are the ones with parallel laser scan strategy and without employing support structures. However, the biggest impact in terms of CRS introduced in the sample comes from the laser speed, as the low laser speed (300 mm/s) lead to notably higher and deeper CRS values. This is in agreement with a previous study on the TRS in LPBF samples with the same build parameters but without LSP treatment, resulting in the same conclusion on the superior LPBF processing parameters^25^. This indicates that there is no further cross-correlation between the LSP and LPBF processing parameters and they can be studied and benchmarked independently.

## Conclusions

The application of neutron Bragg edge imaging enabled an efficient parametric study on a large series of stainless steel 316L samples with varying LPBF and LSP processing parameters. It is concluded that the LSP effects can be characterized in principle independent of the LPBF build parameters, a detailed neutron Bragg edge imaging study, which has earlier been presented elsewhere^25^. LSP treatments were performed with two different laser energies namely 1 J and 1.5 J, as well as with two different laser spot overlaps of 40$\%$ and 80$\%$. It was found that the two laser energies have a much smaller impact on the resulting strain fields than the two chosen overlaps. This, however, appears well justified by the larger effect on the total delivered energy per area by the increased treatment density than by the laser energy increase by just 50$\%$. Correspondingly, the most beneficial effects in terms of induced CRS and CRS depth are found with 1.5 J laser energy and 80$\%$ laser treatment overlap. A further key aspect of the study was the assessment of different LSP treatment strategies from surface treatment towards the possibilities of 3D-LSP treatments with the perspective to design stress fields through a combined LPBF and LSP treatment process. Thus, non-treated samples were compared with surface treated samples as well as samples with a buried treated layer and a combination of a buried layer and surface treatment, referred to as 3D-LSP^11^. In this regards it is found, that in all cases the treatment effects can be well resolved with neutron Bragg edge imaging. A corresponding assessment suggests in particular, that the LSP treatment has to be well optimized in order to provide the envisaged effects in the bulk. This is best underlined by the pronounced and vanishing effects of the 3D-LSP treatment with 1.5 J laser energy but 80$\%$ and 40$\%$ overlap, respectively. For the successful treatment with 80$\%$ overlap the gain in CRS and CRS depth is found to be beyond 50$\%$ and 100$\%$ respectively.
